# A COVID-19 Outbreak in a Rheumatology Department Upon the Early Days of the Pandemic

**DOI:** 10.3389/fmed.2020.576162

**Published:** 2020-09-25

**Authors:** Vasco C. Romão, Filipa Oliveira-Ramos, Ana Rita Cruz-Machado, Patrícia Martins, Sofia Barreira, Joana Silva-Dinis, Luís Mendonça-Galaio, Helena Proença, José Melo Cristino, Ema Sacadura-Leite, Nikita Khmelinskii, José Carlos Romeu, João Eurico Fonseca, The CHULN Rheumatology Department, Manuel António

**Affiliations:** Author Affiliations: Rheumatology Department, Hospital de Santa Maria, Centro Hospitalar Universitário Lisboa Norte, Lisbon Academic Medical Center, Lisbon, Portugal; Rheumatology Research Unit, Instituto de Medicina Molecular João Lobo Antunes, Faculdade de Medicina, Universidade de Lisboa, Lisbon, Portugal; Rheumatology Department, Hospital de Santa Maria, Centro Hospitalar Universitário Lisboa Norte, Lisbon Academic Medical Center, Lisbon, Portugal; Rheumatology Research Unit, Instituto de Medicina Molecular João Lobo Antunes, Faculdade de Medicina, Universidade de Lisboa, Lisbon, Portugal; Rheumatology Department, Hospital de Santa Maria, Centro Hospitalar Universitário Lisboa Norte, Lisbon Academic Medical Center, Lisbon, Portugal; Rheumatology Research Unit, Instituto de Medicina Molecular João Lobo Antunes, Faculdade de Medicina, Universidade de Lisboa, Lisbon, Portugal; Rheumatology Department, Hospital de Santa Maria, Centro Hospitalar Universitário Lisboa Norte, Lisbon Academic Medical Center, Lisbon, Portuga; Rheumatology Research Unit, Instituto de Medicina Molecular João Lobo Antunes, Faculdade de Medicina, Universidade de Lisboa, Lisbon, Portugal; Rheumatology Department, Hospital de Santa Maria, Centro Hospitalar Universitário Lisboa Norte, Lisbon Academic Medical Center, Lisbon, Portugal; Rheumatology Research Unit, Instituto de Medicina Molecular João Lobo Antunes, Faculdade de Medicina, Universidade de Lisboa, Lisbon, Portugal; Rheumatology Department, Hospital de Santa Maria, Centro Hospitalar Universitário Lisboa Norte, Lisbon Academic Medical Center, Lisbon, Portugal; Rheumatology Research Unit, Instituto de Medicina Molecular João Lobo Antunes, Faculdade de Medicina, Universidade de Lisboa, Lisbon, Portugal; Rheumatology Department, Hospital de Santa Maria, Centro Hospitalar Universitário Lisboa Norte, Lisbon Academic Medical Center, Lisbon, Portugal; Internal Medicine 1 Department, Hospital de Santa Maria, Centro Hospitalar Universitário Lisboa Norte, Lisbon Academic Medical Center, Lisbon, Portugal; Rheumatology Department, Hospital de Santa Maria, Centro Hospitalar Universitário Lisboa Norte, Lisbon Academic Medical Center, Lisbon, Portugal; Rheumatology Research Unit, Instituto de Medicina Molecular João Lobo Antunes, Faculdade de Medicina, Universidade de Lisboa, Lisbon, Portugal; Rheumatology Department, Hospital de Santa Maria, Centro Hospitalar Universitário Lisboa Norte, Lisbon Academic Medical Center, Lisbon, Portugal; Rheumatology Department, Hospital de Santa Maria, Centro Hospitalar Universitário Lisboa Norte, Lisbon Academic Medical Center, Lisbon, Portugal; Rheumatology Department, Hospital Garcia de Orta, Almada, Portugal; Rheumatology Department, Hospital de Santa Maria, Centro Hospitalar Universitário Lisboa Norte, Lisbon Academic Medical Center, Lisbon, Portugal; Rheumatology Department, Hospital de Santa Maria, Centro Hospitalar Universitário Lisboa Norte, Lisbon Academic Medical Center, Lisbon, Portugal; Rheumatology Research Unit, Instituto de Medicina Molecular João Lobo Antunes, Faculdade de Medicina, Universidade de Lisboa, Lisbon, Portugal; Rheumatology Department, Hospital de Santa Maria, Centro Hospitalar Universitário Lisboa Norte, Lisbon Academic Medical Center, Lisbon, Portugal; Rheumatology Research Unit, Instituto de Medicina Molecular João Lobo Antunes, Faculdade de Medicina, Universidade de Lisboa, Lisbon, Portugal; Rheumatology Department, Hospital de Santa Maria, Centro Hospitalar Universitário Lisboa Norte, Lisbon Academic Medical Center, Lisbon, Portugal; Rheumatology Department, Hospital de Santa Maria, Centro Hospitalar Universitário Lisboa Norte, Lisbon Academic Medical Center, Lisbon, Portugal; Rheumatology Research Unit, Instituto de Medicina Molecular João Lobo Antunes, Faculdade de Medicina, Universidade de Lisboa, Lisbon, Portugal; Rheumatology Department, Hospital de Santa Maria, Centro Hospitalar Universitário Lisboa Norte, Lisbon Academic Medical Center, Lisbon, Portugal; Division of Rheumatology, Hospital das Clínicas HCFMUSP, Faculdade de Medicina, Universidade de São Paulo, São Paulo, Brazil; Rheumatology Department, Hospital de Santa Maria, Centro Hospitalar Universitário Lisboa Norte, Lisbon Academic Medical Center, Lisbon, Portugal; Rheumatology Department, Hospital de Santa Maria, Centro Hospitalar Universitário Lisboa Norte, Lisbon Academic Medical Center, Lisbon, Portugal; Rheumatology Research Unit, Instituto de Medicina Molecular João Lobo Antunes, Faculdade de Medicina, Universidade de Lisboa, Lisbon, Portugal; Rheumatology Department, Hospital de Santa Maria, Centro Hospitalar Universitário Lisboa Norte, Lisbon Academic Medical Center, Lisbon, Portugal; Rheumatology Research Unit, Instituto de Medicina Molecular João Lobo Antunes, Faculdade de Medicina, Universidade de Lisboa, Lisbon, Portugal; Rheumatology Department, Hospital de Santa Maria, Centro Hospitalar Universitário Lisboa Norte, Lisbon Academic Medical Center, Lisbon, Portugal; Rheumatology Department, Hospital de Santa Maria, Centro Hospitalar Universitário Lisboa Norte, Lisbon Academic Medical Center, Lisbon, Portugal; Rheumatology Research Unit, Instituto de Medicina Molecular João Lobo Antunes, Faculdade de Medicina, Universidade de Lisboa, Lisbon, Portugal; Rheumatology Department, Hospital de Santa Maria, Centro Hospitalar Universitário Lisboa Norte, Lisbon Academic Medical Center, Lisbon, Portugal; Rheumatology Research Unit, Instituto de Medicina Molecular João Lobo Antunes, Faculdade de Medicina, Universidade de Lisboa, Lisbon, Portugal; Rheumatology Department, Hospital de Santa Maria, Centro Hospitalar Universitário Lisboa Norte, Lisbon Academic Medical Center, Lisbon, Portugal; Rheumatology Research Unit, Instituto de Medicina Molecular João Lobo Antunes, Faculdade de Medicina, Universidade de Lisboa, Lisbon, Portugal; Rheumatology Department, Hospital de Santa Maria, Centro Hospitalar Universitário Lisboa Norte, Lisbon Academic Medical Center, Lisbon, Portugal; Rheumatology Research Unit, Instituto de Medicina Molecular João Lobo Antunes, Faculdade de Medicina, Universidade de Lisboa, Lisbon, Portugal; Rheumatology Department, Hospital de Santa Maria, Centro Hospitalar Universitário Lisboa Norte, Lisbon Academic Medical Center, Lisbon, Portugal; Rheumatology Research Unit, Instituto de Medicina Molecular João Lobo Antunes, Faculdade de Medicina, Universidade de Lisboa, Lisbon, Portugal; Rheumatology Department, Hospital de Santa Maria, Centro Hospitalar Universitário Lisboa Norte, Lisbon Academic Medical Center, Lisbon, Portugal; Rheumatology Research Unit, Instituto de Medicina Molecular João Lobo Antunes, Faculdade de Medicina, Universidade de Lisboa, Lisbon, Portugal; Rheumatology Department, Hospital de Santa Maria, Centro Hospitalar Universitário Lisboa Norte, Lisbon Academic Medical Center, Lisbon, Portugal; Internal Medicine Department, Hospital de Faro, Centro Hospitalar Universitário do Algarve, Faro, Portugal; ^1^Rheumatology Department, Hospital de Santa Maria, Centro Hospitalar Universitário Lisboa Norte, Lisbon Academic Medical Center, Lisbon, Portugal; ^2^Rheumatology Research Unit, Instituto de Medicina Molecular João Lobo Antunes, Faculdade de Medicina, Universidade de Lisboa, Lisbon, Portugal; ^3^Occupational Health Department, Hospital de Santa Maria, Centro Hospitalar Universitário Lisboa Norte, Lisbon Academic Medical Center, Lisbon, Portugal; ^4^Clinical Pathology Department, Hospital de Santa Maria, Centro Hospitalar Universitário Lisboa Norte, Lisbon Academic Medical Center, Lisbon, Portugal

**Keywords:** COVID-19, rheumatology practice, rheumatic patients, healthcare workers (HCW), presymptomatic transmission

## Abstract

**Objectives:** To describe our experience with a coronavirus disease 2019 (COVID-19) outbreak within a large rheumatology department early in the pandemic.

**Methods:** Symptomatic and asymptomatic healthcare workers (HCWs) had a naso-oropharyngeal swab for detection of severe acute respiratory syndrome coronavirus 2 (SARS-CoV-2) and were followed clinically. Reverse transcription polymerase-chain reaction (RT-PCR) was repeated to document cure, and serological response was assessed. Patients with risk contacts within the department in the 14 days preceding the outbreak were screened for COVID-19 symptoms.

**Results:** 14/34 HCWs (41%; 40 ± 14 years, 71% female) tested positive for SARS-CoV-2, and 11/34 (32%) developed symptoms but were RT-PCR-negative. Half of RT-PCR-positive HCWs did not report fever, cough, or dyspnea before testing, which were absent in 3/14 cases (21%). Mild disease prevailed (79%), but 3 HCWs had moderate disease requiring further assessment, which excluded severe complications. Nevertheless, symptom duration (28 ± 18 days), viral shedding (31 ± 10 days post-symptom onset, range 15–51), and work absence (29 ± 28 days) were prolonged. 13/14 (93%) of RT-PCR-positive and none of the RT-PCR-negative HCWs had a positive humoral response Higher IgG indexes were observed in individuals over 50 years of age (14.5 ± 7.7 vs. 5.0 ± 4.4, *p* = 0.012). Of 617 rheumatic patients, 8 (1.3%) developed COVID-19 symptoms (1/8 hospitalization, 8/8 complete recovery), following a consultation/procedure with an asymptomatic (7/8) or mildly symptomatic (1/8) HCW.

**Conclusions:** A COVID-19 outbreak can occur among HCWs and rheumatic patients, swiftly spreading over the presymptomatic stage. Mild disease without typical symptoms should be recognized and may evolve with delayed viral shedding, prolonged recovery, and adequate immune response in most individuals.

## Introduction

Following the initial descriptions in early January 2020 of a novel form of severe pneumonia in patients from Wuhan, China (1–4), the coronavirus disease 2019 (COVID-19) quickly spread at a global level. On January 30, the World Health Organization declared it a public health emergency of international concern (5), and it was subsequently updated to a pandemic on March 11 (6). After the first reported case in Portugal (March 2), exponential growth led to the institution of major restrictive measures (7).

Consistently high infection rates among healthcare workers (HCWs) have been reported in several hard-hit countries, such as China (8, 9), Italy (10), Spain (11), and the United States (12), despite adequate safety measures (13). One possibility is that in-hospital transmission among patients and HCWs might be a key form of contagion (9, 14, 15). This is particularly relevant given the transmission dynamics of severe acute respiratory syndrome coronavirus 2 (SARS-CoV-2), whereby presymptomatic/asymptomatic contamination is likely to play a major role in disease spreading (14–19).

In the early days of the pandemic, most focus was given to severe clinical pictures (2, 8, 9, 20), and reports on mild or asymptomatic disease were scarce (21, 22). This may have contributed to an initial oversight of more general, less severe manifestations, such as upper respiratory and digestive symptoms (23). These milder disease forms might be easily undervalued, including by HCWs responding to the pandemic. In healthcare facilities, this may facilitate the generalized spread among HCWs, who can serve as disease-transmission agents (9, 14–16). This fact may be particularly relevant in outpatient-oriented departments with a high volume of clinical activity (e.g., rheumatology). In addition, rheumatology practice requires daily close physical contact with patients with rheumatic and musculoskeletal diseases (RMDs), who are often immunosuppressed and have an increased overall infectious risk.

In the present report, we aim to describe our experience with a COVID-19 outbreak within our department, upon the initial weeks of the pandemic, highlighting clinical, virological, and immunological outcomes of HCWs and RMD patients.

## Materials and Methods

### Outbreak Characterization

Over the week of March 9–15, 2020, several HCWs of the rheumatology department of Centro Hospitalar Universitário Lisboa Norte (CHULN) developed mild symptoms compatible with COVID-19. All staff (symptomatic/asymptomatic) underwent screening for SARS-CoV-2 on March 15–16. Double naso-oropharyngeal swabs were obtained, and samples were tested for SARS-CoV-2 by reverse transcription-polymerase chain reaction (RT-PCR; cobas®SARS-CoV-2 kit, cobas®6800 System, Roche Diagnostics, USA). All the confirmed and suspected cases were quarantined and referred to public health authorities. Daily remote clinical monitoring of HCWs was conducted by 2 asymptomatic rheumatologists in conjunction with public health and occupational medicine specialists. Testing of HCWs was repeated (i) 7–14 days after the first negative test in subjects with persisting symptoms and (ii) 5–7 days following the resolution of fever and improvement in respiratory symptoms in confirmed cases (24). Two consecutive negative tests were required to confirm viral shedding cessation and allow return to work (25). Immunological response to SARS-CoV-2 was evaluated by chemiluminescent immunoassay (MAGLUMI®800 CLIA System, MAGLUMI®2019-nCoV (SARS-CoV-2) IgM/IgG-kits, Snibe Co., Ltd., China) in all HCWs, following symptom resolution and double-negative RT-PCR in confirmed cases.

We contacted patients observed during the previous 2 weeks in the day-care unit, outpatient clinic, and procedures room who had possible contacts with confirmed RT-PCR-positive HCWs. Each patient was screened for suggestive symptoms and requested to remain in isolation for 14 days post-contact with the department. Patients with symptoms compatible with COVID-19 were referred to the national health system hotline and signaled to health authorities, who had also received the list of screened patients.

### Study Procedures

All HCWs of the rheumatology department who were working during March 2–13, 2020, including visiting fellows, were invited to participate in this study. A standardized questionnaire was administered to collect demographic data, symptom characterization, disease course and outcome, treatment, comorbidities, and concomitant therapy. Results of laboratory and imaging studies performed, including RT-PCR and IgG/IgM for SARS-CoV-2 were reviewed. Disease course was classified as mild, moderate (requiring physical examination and laboratory/imaging studies), or severe (requiring hospitalization). Moreover, patients observed in the department between March 2–13 who developed symptoms suggestive of COVID-19 had an appointment scheduled, upon definite resolution, for clinical observation. The same data were collected as for HCWs, in addition to variables related to the RMD and associated treatment. Patients observed in the period of interest who did not develop COVID-19 symptoms or did so outside the 14-day window after the last contact with the department, were excluded. All study participants signed a study-specific informed consent. This study was approved by the Lisbon Academic Medical Center Ethics Committee (reference 171/20).

### Statistical Analysis

Demographic and clinical characteristics were presented as frequency, mean ± standard deviation, or median [interquartile range (IQR)] as applicable. Comparison of continuous variables between HCW groups was performed using Kruskal–Wallis (3 groups) or Mann–Whitney U-test (2 groups). Categorical variables were compared using Chi-square or Fisher's exact test. Agreement between RT-PCR and serological tests was done using Kappa statistic. Pearson correlation was applied to study the relation of IgG humoral response and clinical variables. Statistical analyses were performed using Stata-12.1 for Mac (StataCorp, College Station, USA) and GraphPad-Prism-7 for MacOS (GraphPad Software, USA). *P*-value was considered significant at *p* < 0.05.

## Results

### Clinical and Virological Course of HCWs

**Figure 1:**
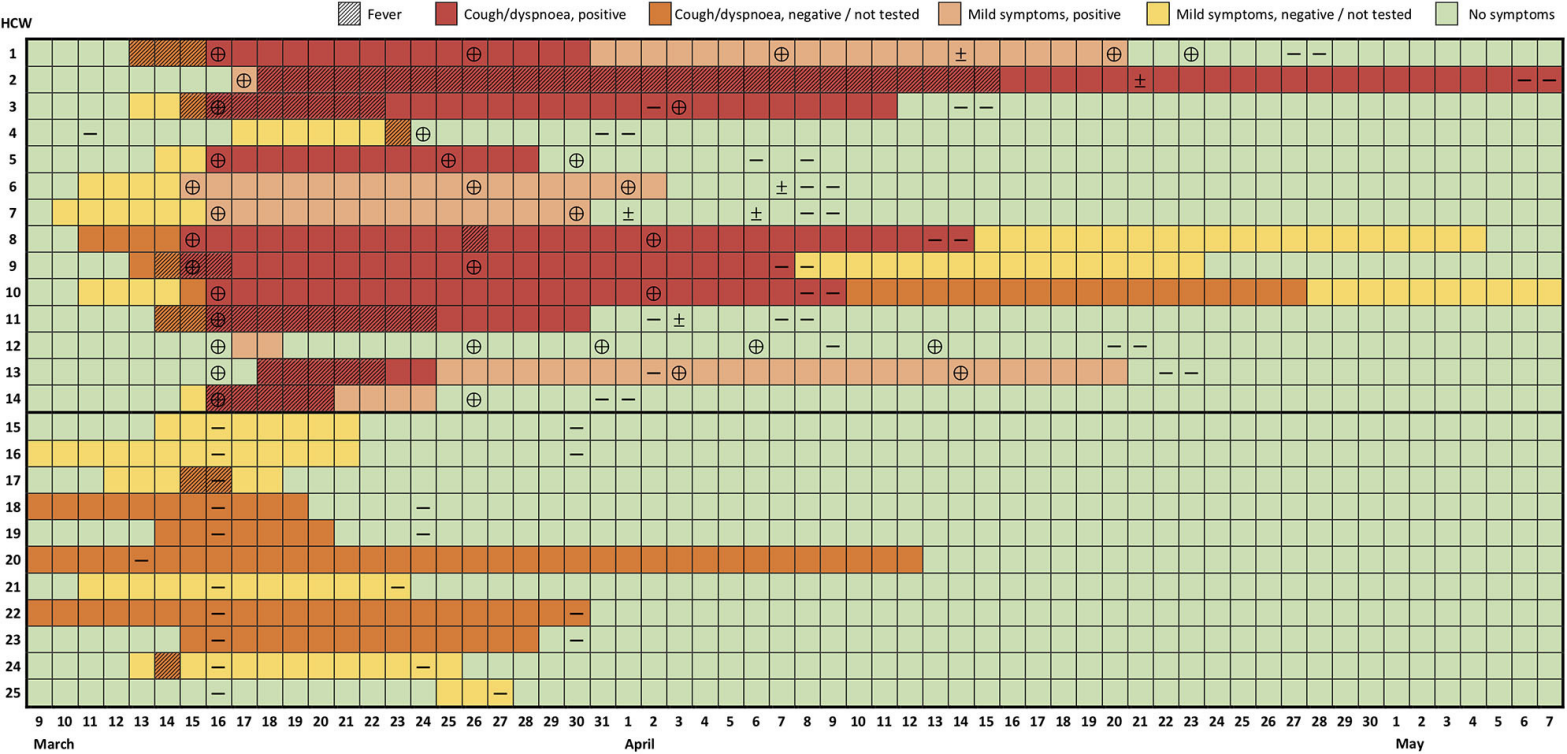
Evolution of symptoms and RT-PCR test results of healthcare workers with confirmed or suspected COVID-19. Each row represents a healthcare worker (HCW) followed over time (columns). Results of reverse transcription polymerase-chain reaction (RT-PCR) are indicated as positive (⊕), negative (—) or indeterminate (±). Mild symptoms include symptoms other than fever, cough, and dyspnea as referred to in the text and Table 1.

A total of 25/34 HCWs (17 rheumatologists, 8 residents, 4 visiting fellows, 1 nurse, 1 health aid, 2 secretaries, and 1 cleaning aid) developed symptoms suggestive of a viral infection, 14 of whom had a positive RT-PCR for SARS-CoV-2 (Table 1, Figure 1, Supplementary Figure 1). Ten out of 14 (71%) positive cases were female or younger than 50 years old. Only 4/14 (29%) subjects had a previous history of cardiovascular disease and/or metabolic syndrome, whereas 3/14 (21%) had a diagnosis of immune-mediated inflammatory disease (one of whom treated with methotrexate 15 mg/week). Importantly, 5/14 (36%) HCWs did not develop fever, which lasted ≤3 days in 4/9 (44%) remaining cases. Cough was also absent in the same proportion (36%). Of note, 7/14 (50%) subjects did not develop any of the manifestations of the typical COVID-19 triad prior to the positive RT-PCR test, which were completely missing in 3 cases (21%) throughout the disease. In turn, milder symptoms were already present during the week prior to the outbreak identification in several instances. Anosmia and dysgeusia were present in over half the cases, including 1 subject (HCW6) who did not develop fever, cough, or dyspnea.

**Table 1:** Demographic and clinical characteristics of study participants.

	**Overall (*n* = 34)**	**SARS-CoV-2 RT-PCR test results and symptoms**			***p*-value**
		**Positive (*n* = 14)**	**Negative, symptoms**	**Negative, no symptoms**	
			**(*n* = 11)**	**(*n* = 9)**	
Age, years (range)	41 ± 12 (26–66)	40 ± 14 (26–62)	40 ± 8 (28–52)	44 ± 15 (27–66)	0.759
Age ≥ 60 years, *n* (%)	4 (11.8)	2 (14.3)	0	2 (22.2)	0.286
Female, *n* (%)	23 (67.7)	10 (71.4)	8 (72.7)	5 (55.6)	0.723
Comorbidities, *n* (%)	20 (58.8)	8 (57.1)	6 (54.6)	6 (66.7)	0.849
Arterial hypertension	5 (14.7)	3 (21.4)	0	2 (22.2)	0.279
Cardiac disease	3 (8.8)	2 (14.3)	0	1 (11.1)	0.601
Diabetes mellitus	2 (5.9)	2 (14.3)	0	0	0.324
Obesity	3 (8.8)	2 (14.3)	1 (9.1)	0	0.768
COPD/asthma	3 (8.8)	1 (7.1)	2 (18.2)	0	0.464
Chronic rhinosinusitis	4 (11.8)	1 (7.1)	2 (18.2)	1 (11.1)	0.806
IMID	6 (17.7)	3 (21.4)	1 (9.1)	2 (22.2)	0.732
Cancer history	2 (5.9)	0	1 (9.1)	1 (11.1)	0.241
Smoking history (ever)	9 (26.5)	4 (28.6)	2 (18.2)	3 (33.3)	0.790
Concomitant therapy, *n* (%)	5 (14.7)	3 (21.4)	0	2 (22.2)	0.279
ACEi/ARB	5 (14.7)	3 (21.4)	0	2 (22.2)	0.279
NSAIDs	1 (2.9)	0	1 (9.1)	0	0.588
DMARDs	3 (8.8)	1 (7.1)	1 (9.1)	1 (12.5)	1.000
COVID-19 symptoms, *n* (%)/duration, days	25 (73.5)/24 ± 19	14 (100)/28 ± 18	11 (100)/20 ± 20	0	–
Fever	11 (32.4)/6 ± 8	9 (64.3)/7 ± 9	2 (18.2)/2 ± 1	–	0.042
Cough	15 (44.1)/25 ± 19	9 (64.3)/25 ±14	6 (54.6)/26 ± 26	–	0.622
Dyspnea	3 (8.8)/3 ± 31	2 (14.3)/14 ±10	1 (9.1)/66		1.000
Chest tightness	4 (11.8)/17 ± 16	3 (21.4)/22 ±16	1 (9.1)/4	–	0.603
Malaise	10 (29.4)/15 ± 14	9 (64.3)/16 ± 15	1 (9.1)/6	–	0.012
Fatigue	12 (35.3)/25 ± 21	10 (71.4)/24 ± 21	2 (18.2)/27 ± 25	–	0.015
Headache	11 (32.4)/16 ± 18	10 (71.4)/17 ± 19	1 (9.1)/5	–	0.004
Rhinorrhea	14 (41.2)/18 ± 16	9 (64.3)/24 ± 17	5 (45.5)/7 ± 3	–	0.435
Sore throat	16 (47.1)/7 ± 7	7 (50.0)/9 ± 9	9 (81.8) 5 ± 3	–	0.208
Anosmia	9 (26.5)/15 ± 13	8 (57.1)/16 ± 13	1 (9.1)/6	–	0.033
Dysgeusia	8 (23.5)/12 ± 7	8 (57.1)/12 ± 7	0	–	0.003
Arthralgia	1 (2.9)/25	1 (7.4)/25	0	–	1.000
Myalgia	11 (32.4)/14 ± 17	8 (57.1)/16 ± 20	3 (27.3)/8 ± 9	–	0.004
Abdominal pain	2 (5.9)/3 ± 1	0	2 (18.2)/3 ± 1	–	0.183
Nausea/vomiting/diarrhea	7 (20.6)/11 ± 11	5 (35.7)/13 ± 12	2 (18.2)/5 ± 0	–	0.407
Dizziness	3 (8.8)/9 ± 6	2 (14.3)/10 ± 8	1 (9.1)/8	–	1.000
Disease severity, *n* (%)					
Mild	22 (64.7)	11 (78.6)	11 (100)	–	0.230
Moderate	3 (8.8)	3 (21.4)	0		
Severe	0	0	0		
Hospitalization	0	0	0	–	–
Treatment, *n* (%)/duration, days					
None/supportive	27 (79.4)	7 (50)	11 (100)	9 (100)	–
Hydroxychloroquine	7 (20.6)/9 ± 3	7 (50)/9 ± 3	0	0	
Azithromycin	4 (11.8)/5 ± 0	4 (28.6)/5 ± 0	0	0	
Complications, *n* (%)	1 (2.9)	1 (7.4)	0	0	–
Symptom resolution, *n* (%)	23 (92)	12 (85.7)	11 (100)	–	–
Secondary transmission, *n* (%)	7 (20.6)	7 (50)	–	–	–
Days to 2 negative tests (range)	31 ± 10 (15–51)	31 ± 10 (15–51)	–	–	–

*ACEi, angiotensin-converting enzyme inhibitors; ARB, angiotensin-II receptor blockers; chronic obstructive pulmonary disease; DMARDs, disease-modifying anti-rheumatic drugs; IMID, immune-mediated inflammatory disease; NSAIDs, non-steroidal anti-inflammatory drugs. Continuous values represented as mean ± SD*.

The majority of cases (11/14, 79%) had a benign course. There were no hospitalizations, but 3/14 HCWs (aged 45–61 with relevant comorbidities) underwent clinical, laboratory, and radiographic evaluation 7–12 days after symptom onset due to persistent fever, cough, chest pain, and/or shortness of breath (Supplementary Table 1). Lymphopenia (1/3), thrombocytopenia (1/3), raised lactate dehydrogenase (1/3), D-dimers (2/3), fibrinogen (2/3), and C-reactive protein (CRP; 2/3) were identified, but hypoxemia and radiographic signs of COVID-19 pneumonia were absent. Seven subjects (50%) were treated with hydroxychloroquine (400 mg/day, median 9 days, range 7–14 days), and 4 received concomitant azithromycin (500 mg/day, 5 days). One HCW developed a bacterial sinus infection, treated with amoxicillin/clavulanate. Secondary transmission to household members was confirmed in 7/14 (50%) cases, one to 2 close relatives, all with mild disease.

Despite the favorable course of most cases, symptom duration was prolonged (median 24.5 days, IQR 15–39, range 2–58; Table 1, Figure 1, Supplementary Figure 1). At the end of follow-up, 2 subjects had persistent symptoms (Figure 1, Supplementary Figure 1). Likewise, naso-oropharyngeal RT-PCR remained positive on average for 31 ± 10 days from symptom onset (median 29.5 days, IQR 25–35, range 15–51; Figure 1). This resulted in the need to repeat RT-PCR tests frequently, with a median number of tests per positive subject of 5 (IQR 4–6, range 4–9). Of note, 8/34 (24%) of repetition tests in completely asymptomatic subjects were positive. Yet, this was less than in individuals who repeated testing while still showing some symptoms (13/25, 52%, *p* = 0.024; Figure 1). On average, HCWs were away from work for 29.2 ± 9.8 days (median 24.5, IQR 23–36, range 16–51).

Eleven subjects developed various symptoms but tested negative even upon retesting (Table 1, Figure 1). These HCWs reported complaints of cough (55%), rhinorrhea (45%), sore throat (82%), and other symptoms in similar frequency and duration to confirmed cases over the same time frame. However, fever, fatigue, malaise, headache, myalgia, anosmia, and dysgeusia were significantly less common. Notably, these HCWs had a comparable demographic and comorbidity profile to those with positive RT-PCR and the 9 asymptomatic subjects with negative RT-PCR (Table 1).

No HCWs reported travel from areas with active community transmission. A resident (HCW6) wearing a surgical mask observed a suggestive case in the emergency department 3 days before symptom onset (March 8), who did not fulfill testing criteria at the time (travel from endemic area). A consultant (HCW4) had a short, unprotected contact in the week preceding the outbreak with an inpatient from another department who was later found to have COVID-19. Of note, 7/14 of infected HCWs had a common link to our rheumatological procedures unit, having spent the most hours there over the previous 2 weeks. Nonetheless, the remaining RT-PCR-positive HCWs had minimal exposure to this facility, and 2 rheumatologists (HCW18/21), who spent more than 10 h/week in the unit, tested negative. Finally, all but 10 HCWs (5 RT-PCR-positive, 5 RT-PCR-negative) were present, unprotected, in a 2.5-h departmental meeting (March 10) addressing the local response to the pandemic. At the time, only 1 HCW (HCW7) had symptoms (mild rhinorrhea).

### Immunological Response

**Figure 2:**
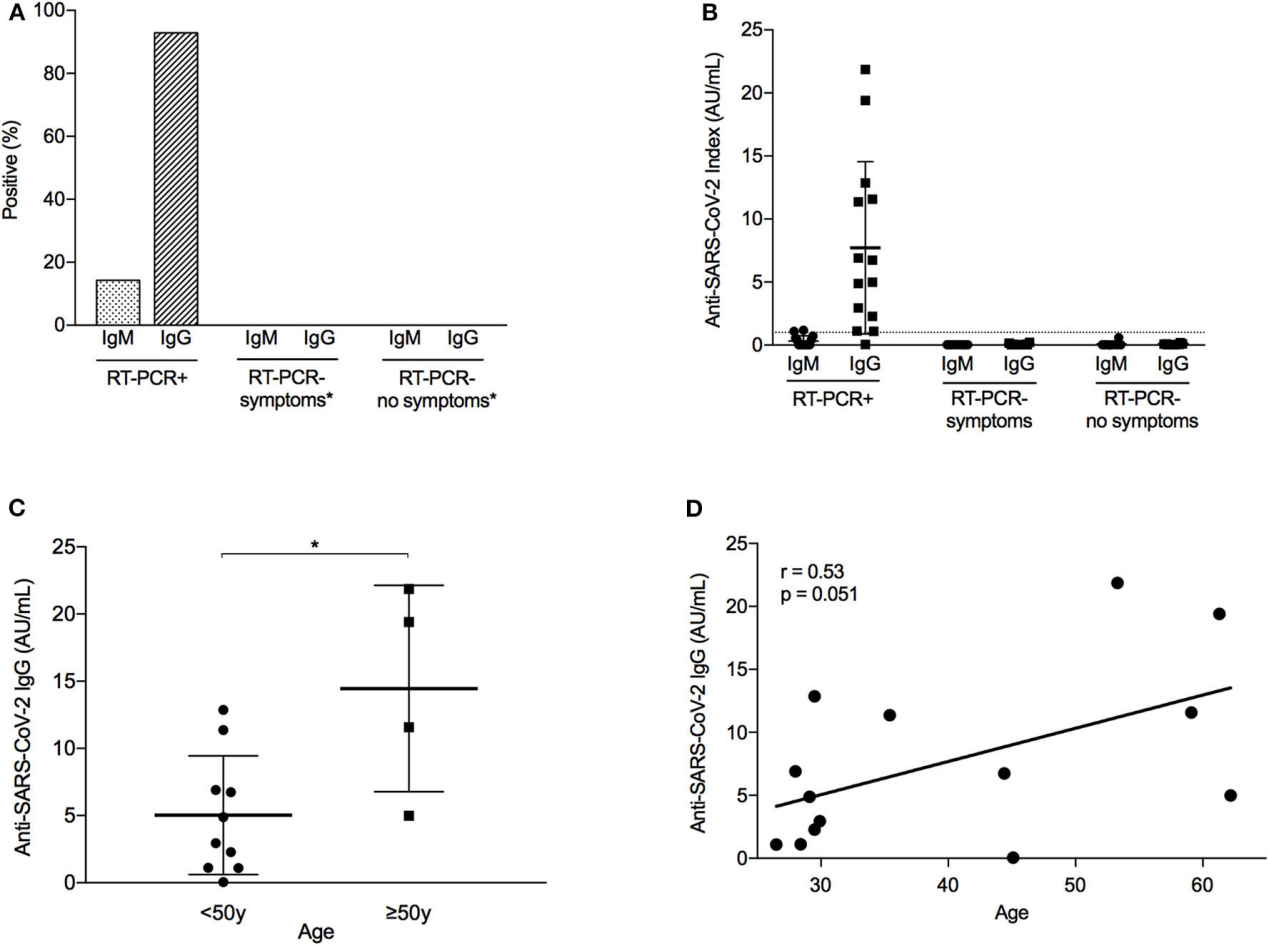
Immunological response to SARS-CoV-2 infection among healthcare workers. **(A)** Percentage of healthcare workers (HCWs) with a positive immunological response to severe acute respiratory syndrome coronavirus 2 (SARS-CoV-2) infection, defined as an IgM or IgG index equal or above 1.0 AU/mL. Analysis differentiated by HCW group, depending on the presence of COVID-19 symptoms and the result of reverse transcription-polymerase chain reaction (RT-PCR). *N* = 32 (RT-PCR+, *N* = 14; RT-PCR– symptoms, *n* = 10; RT-PCR– no symptoms, *n* = 8). *None of the HCWs in this group developed anti-SARS-CoV-2 antibodies (i.e., frequency = 0%) **(B)** Index of anti-SARS-CoV-2 antibodies (IgG and IgM) according to RT-PCR result and COVID-19 symptoms. Dashed line represents positive threshold (1.0 AU/mL). Error bars represent mean with standard deviation. **(C)** Distribution of anti-SARS-CoV-2 IgG antibodies in RT-PCR+ HCWs, according to age group (below or above 50 years old). Error bars represent mean with standard deviation. *p = 0.012. **(D)** Correlation between age and anti-SARS-CoV-2 IgG antibodies in RT-PCR+ HCWs. *r*, Pearson correlation coefficient.

After a median (IQR) of 45 (40.5–48.5) days following symptom onset (or the first RT-PCR test for asymptomatic subjects), 32 HCWs had an assessment of the serological response (Figure 2). A positive IgM and IgG index (>1.0 AU/mL) was seen in, respectively, 2/14 (14.3%) and 13/14 (92.9%) of the confirmed RT-PCR-positive cases and none of the symptomatic/asymptomatic RT-PCR-negative subjects (Figures 2A,B). Both tests had a 96.9% agreement in case classification (Kappa coefficient 0.936). Assessment timing was similar for the HCWs with borderline positive IgM (HCW 11/12, 1.10–1.18 AU/mL) or IgG (HCW 12/14, 1.10 AU/mL) compared to other RT-PCR-positive subjects. In addition, HCW10 had an IgG index below the positive threshold, despite 2 positive RT-PCR tests, no immunosuppression, and comparable evaluation timing and clinical course.

Within the RT-PCR-positive group, considerable variation was seen in the antibody response (Figure 2B). Notably, subjects over 50 years old had a higher mean IgG index (14.5 ± 7.7 AU/mL) than younger individuals (5.0 ± 4.4 AU/mL, *p* = 0.012; Figure 2C). Although the numbers are small, the 3 older HCWs (HCW 8/11/13) who had an IgG index above 10 AU/mL experienced a more severe disease course with high fever and cough, and 2 of them had raised D-dimers, fibrinogen, and CRP. In contrast, the remaining older HCW (HCW 7) had a mild course with limited rhinorrhea and gastrointestinal symptoms and developed a lower IgG index (4.99 AU/mL). Nevertheless, a positive trend was observed in the correlation between age and IgG index (Pearson *r* = 0.53, *p* = 0.051; Figure 2D). No other clinical factor was associated with antibody response, including sex; treatment; or presence/duration of fever, cough, or dyspnea.

### Secondary Transmission to Patients With RMDs

**Figure 3:**
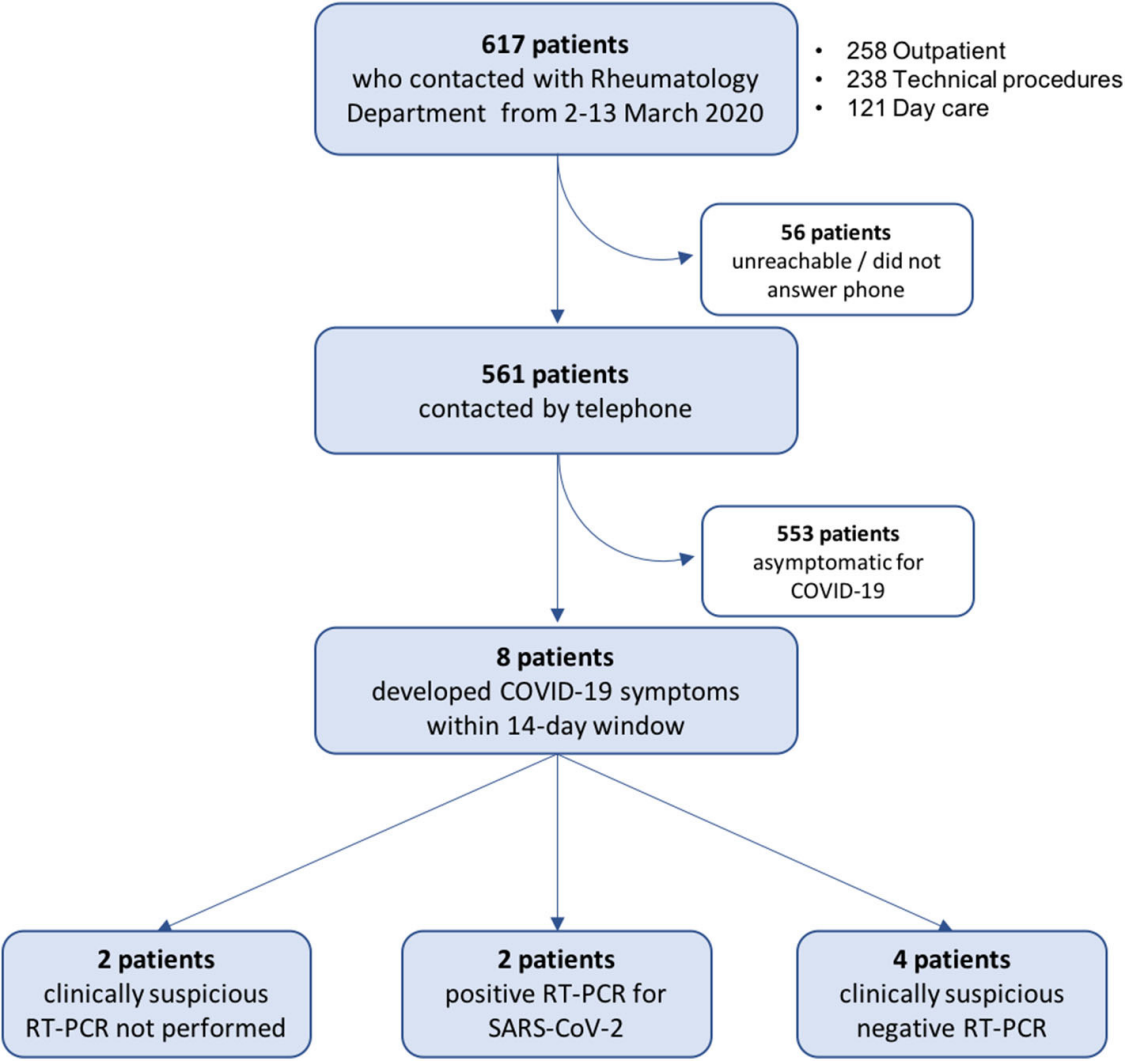
Flow chart of RMD patient screening for symptoms suggestive of COVID-19. Patients with possible contacts with healthcare workers with positive reverse transcription-polymerase chain reaction (RT-PCR) for severe acute respiratory coronavirus 2 (SARS-CoV-2) in the period of March 2–13, 2020, were contacted by telephone and screened for COVID-19 symptoms starting within the subsequent 14 days. See Methods for details.

A total of 617 patients were identified as having had a potential risky contact, 561 (91%) of whom were contacted by telephone and screened for COVID-19 symptoms starting within the 14-day window (Figure 3). We identified 8 (1.3% of total) female patients (mean age 66.8 ± 14.9 years) who developed symptoms compatible with COVID-19 (Table 2). Six patients had a diagnosis of an inflammatory RMD; 3 were treated with conventional synthetic disease-modifying antirheumatic drugs (csDMARDs) and glucocorticoids and 2 with biologic DMARDs (bDMARDs). All contacts took place within the same 2 days (March 9 and 11), all but one were with a confirmed infected HCW, and patients denied additional suspicious contacts. Contact tracing for Patient 1 within the department confirmed it to be limited to a symptomatic physician with negative RT-PCR and serology (HCW24). Importantly, in 7/8 cases, the HCW was asymptomatic at the time of contact, and 1 patient (Patient 6) had a consultation with a physician (HCW7) presenting only mild serous rhinorrhea. Of note, 5/8 contacts were in the context of diagnostic (ultrasound) or therapeutic procedures (mesotherapy), which involved prolonged close physician–patient contact.

**Table 2:** Clinical features of RMD patients with confirmed or suspected COVID-19.

**Patient ID**	**1**	**2**	**3**	**4**	**5**	**6**	**7**	**8**
Age/Sex	52/F	90/F	68/F	55/F	83/F	70/F	46/F	68/F
Rheumatic disease	RA/SLE overlap	GCA	Viral reactive arthritis (resolved)	APS	Sarcoidosis	RA	PsA	Rotator cuff tendinopathy
Disease duration	11.1 y	10.1 y	7.7 mo	2.2 y	13.7 y	4.7 mo	15.7 y	11.2 mo
Disease activity	Low	Remission	Remission	No events	Remission	Low	Moderate	Low/mild
Comorbidities	HT, heart dx, COPD	HT, CV dx, OP	HT, COPD	HT, CV dx, COPD	HT, heart dx, ILD, CKD	Dyspepsia, spherocytosis	N/A	HT, uterine cancer (past)
Smoking status	Past	Past	Past	Active	Never	Never	Never	Never
DMARDs (dose, mg)	AZT (150), LEF (20)	MTX (12.5/w), denosumab (60/6 mo)	No	No	No	MTX (15/w), HCQ (400)	IFX (3/kg)	No
Glucocorticoids (dose, mg)	DFZ (9)	PDN (5)	No	No	No	PDN (7.5)	No	No
NSAIDs	No	No	Nimesulide	No	Acemetacin	No	Acemetacin	No
ACEi/ARB	Ramipril	No	No	Perindopril	Ramipril	No	No	Lisinopril
HCW contact (#)	RT-PCR– (24)	RT-PCR+ (1)	RT-PCR+ (1)	RT-PCR+ (14)	RT-PCR+ (1)	RT-PCR+ (7)	RT-PCR+ (1)	RT-PCR+ (1)
Contact date	9/3/2020	11/3/2020	9/3/2020	9/3/2020	9/3/2020	11/3/2020	11/3/2020	11/3/2020
Contact type	US (shoulder)	Mesotherapy (shoulder)	Consultation	Consultation	Mesotherapy (lumbar spine)	Consultation	Mesotherapy (heel)	Mesotherapy (shoulder)
Contact duration	15 min	10 min	30 min	30 min	10 min	30 min	10 min	10 min
Days from contact to symptom onset	9	2	4	4	4	3	4	4
COVID-19 symptoms (order of appearance)	(1) Fv, Mal, Ftg, Hdx, Rhin, Cgh; (2) Ansm, Dysg	(1) Mal, Ftg. Myalg, Arth, Cgh; (2) Fv, Hdx, Rhin; (3) Chx, Dysp, Ansm, Dysg	(1) Mal, Ftg, Hdx, Thr, Cgh, Arth; (2) Ansm, Dysg; (3) GI, Dizz	(1) Mal, Hdx, Rhin, Myalg. GI; (2) Cgh, Ftg; (3) Ansm, Dysg	(1) Fv, Mal, Fat, Hdx, Rhin, Arth, Myalg, Dizz	(1) Abd, GI; (2) Rhin, Cgh; (3) Chx, Hdx	(1) Ftg, Cgh; (2) Dysg (3) Hdx, Abd	(1) Mal, Ftg, Hdx, Thr, Cgh; (2) Abd, GI; (3) Fv, Myalg, Dizz
Diagnostic RT-PCR test (symptom day)	Positive (13)	Positive (4)	Inconclusive (11) Negative (21)	Negative (9)	N/A	Negative (8)	N/A	Negative (8)
Symptom duration	14	20	30	21	31	25	29	28
Hospitalization (days)	No	Yes (10)	No	No	No	No	No	No
ICU admission	N/A	No	N/A	N/A	N/A	N/A	N/A	N/A
Complications	No	No	No	No	No	No	No	No
Targeted treatment	No	HCQ, LOP+RIT	No	No	No	No	No	No
Treatment changes	↓ DFZ 6 mg	Suspended MTX	No	No	↑ NSAID freq	No	No	No
Outcome	Cure	Cure	Cure	Cure	Cure	Cure	Cure	Cure
Days to 2 negative tests	34	20	N/A	N/A	N/A	N/A	N/A	N/A

*Abd, abdominal pain; ACEi, angiotensin-converting enzyme inhibitors; Ansm, anosmia; APS, antiphospholipid syndrome; ARB, angiotensin-II receptor blockers; Arth, arthralgia; AZT, azathioprine; Chx, chest pain/tightness; Cgh, cough; CKD, chronic kidney disease; COPD, chronic obstructive pulmonary disease; COVID-19, coronavirus disease 2019; CV, cerebrovascular; dx, disease; DFZ, deflazacort; Dizz, dizziness/vertigo; DMARDs, disease-modifying anti-rheumatic drugs; Dysg, dysgeusia; Dysp, dyspnoea; Freq, frequency; Ftg, fatigue; Fv, fever; GCA, giant cell arteritis; GI, gastrointestinal, including nausea, vomiting, diarrhea; HCQ, hydroxychloroquine; HCW, healthcare worker; Hdx, headache; HT, hypertension; ICU, intensive care unit; IFX, infliximab; ILD, interstitial lung disease; LEF, leflunomide; LOP, lopinavir; Mal, malaise; mo, months; MTX, methotrexate; N/A, not applicable; NSAIDs, non-steroidal anti-inflammatory drugs; OP, osteoporosis; PDN, prednisolone; PsA, psoriatic arthritis; RA, rheumatoid arthritis; RIT, ritonavir; Rhin, rhinorrhea; RMD, rheumatic and musculoskeletal diseases; RT-PCR, reverse transcription-polymerase chain reaction; SLE, systemic lupus erythematosus; Thr, sore throat; US, ultrasound; w, weeks; y, years*.

Patients developed symptoms on average 4.3 ± 2.1 days (range 2–9) after the contact. Half reported fever, 88% had cough, and only 1 patient reported dyspnea. General and upper airway symptoms were common, including anosmia (50%) and dysgeusia (63%). Nasopharyngeal swabs were performed in 6/8 cases (8.8 ± 3.1 days post-symptom onset), 2 of which were positive for SARS-CoV-2, and 1 was inconclusive. Two patients were not tested due to difficulty in reaching health authorities or personal choice (self-isolation). Patients with negative/unavailable tests still had suggestive COVID-19 symptoms.

All but one patient had a mild-to-moderate course and were clinically recovered after an average of 24.8 ± 5.9 days. A 90-year-old woman with giant cell arteritis and relevant cardiovascular comorbidities, exposed to long-term methotrexate and low-dose glucocorticoids, was hospitalized after 6 days of fever and 3 days of worsening chest pain and dyspnea. She required oxygen therapy, received a combination of hydroxychloroquine (400 mg/day) and lopinavir/ritonavir (800/200 mg/day), and was discharged after 10 days. Importantly, none of the patients experienced a flare of the baseline RMD.

## Discussion

Our study provides important lessons on the vulnerability and impact of a COVID-19 outbreak within a large rheumatology department at a time when universal surgical mask use was not recommended. Over a single week, 41% of HCWs were confirmed to be infected by SARS-CoV-2, and an additional 32% developed mostly overlapping symptoms. Although we could not detect the index case, the spread of the contagion was fast and occurred when almost all HCWs were asymptomatic or exhibited only minor symptoms, easily dismissed or attributed to another concurrent viral disease. These findings are in accordance with current concerns around the presymptomatic or asymptomatic transmission of SARS-CoV-2 among HCWs and patients (9, 14–16). As viral shedding and infectiousness are higher in the 2–3 days prior to symptom onset and rapidly decrease thereafter (26, 27), a high proportion of contagion occurs during the presymptomatic stage (18, 26). In addition, asymptomatic (17, 22, 28, 29) and mild disease forms with limited upper respiratory symptoms are now widely recognized (23, 27) and may escape vigilance protocols, more focused on the presence of fever, cough, and dyspnea. This was certainly the case in our cluster, in which testing of all HCWs of the department, whether symptomatic or not, was vital to identify cases and contain the outbreak. Therefore, in healthcare settings, continuous mask use, social distancing, and mild-symptom monitoring should be adopted among HCWs, together with proactive testing strategies, to account for potential pre/asymptomatic carriers (15).

The outbreak had a profound repercussion in the clinical activity of the department. Infected subjects had protracted symptoms and were away from work for around 1 month. In addition, prolonged viral shedding (up to 51 days) led to frequent RT-PCR repetition (median 5 tests) until cure was confirmed, consuming substantial resources. Our findings regarding viral RNA swab positivity are longer than previously reported (20, 30–32), which may be related to differences in specimen collection (double naso- and oropharyngeal swab in our study), study population, or disease severity. Interestingly, similar nasopharyngeal viral loads in patients with mild and severe disease have been reported (30) although a separate study concluded otherwise (33). Moreover, some data suggest that a positive RT-PCR does not denote the actual presence of viable virus, especially after the first week (27, 34). However, this is not yet fully established, and we would, therefore, advocate for 2 consecutive negative tests before HCWs return to work. In effect, 21/59 (36%) of repeat tests were positive, and in 5 instances, a positive or indeterminate test followed a first negative result, highlighting the difficulties of interpretation (34).

All HCWs had a mild-to-moderate disease course, and there were no major complications. We believe the positive outcome of the cohort is mainly related to the young mean age with only 2 HCWs older than 60 years. Alternatively, a lower initial viral exposure load could also explain an overall milder phenotype (33). Nevertheless, most of the infected HCWs (93%) developed an immune response, which tended to be more robust in older individuals. This, in turn, may be secondary to a more severe clinical course, known to be strongly associated with age (20, 35). A possible explanation for this finding could be a higher peak viral load, also previously shown to be positively correlated with age (30). Indeed, we highlight that 2/13 (15.4%) IgG-positive HCWs, both under 30 years old and with a very mild disease course, had borderline IgG indexes. Nevertheless, other factors, such as T-cell–mediated immunity (36–38), may be involved, as 1 HCW in the 40- to 50-year-old range who had cough and 2 positive RT-PCR tests did not develop IgG antibodies 47 days post-symptom onset. Concordance between serology and RT-PCR was otherwise excellent, confirming previous reports (20, 30, 31). Although it cannot be completely excluded, this suggests there were no false-negative RT-PCR results, including in symptomatic HCWs.

Finally, secondary transmission to a minority of patients did occur from 4 HCWs who were asymptomatic (75%) or had mild upper airway symptoms (25%), mostly in close proximity contact. As one of the confirmed cases only contacted with HCW 24 (negative RT-PCR and serology), we cannot exclude undisclosed community contagion or nosocomial transmission through fomites (39). Also, we admit that patients with negative/missing RT-PCR could be false negative or undiagnosed cases, possibly due to a larger interval between symptom onset and testing. Of note, all contacts occurred when preventive measures had already been adopted, and 80% of face-to-face clinical activity had been deferred, which might explain the low number of infected patients. We admit the possibility that contagion could have followed the opposite route (pre/asymptomatic patients to HCWs) although symptom timing does suggest otherwise. Notwithstanding the advanced age and long-term use of cs/bDMARDs and low-dose glucocorticoids in half the cases, all patients had a favorable outcome. This is in accordance with recent data that did not demonstrate an increased incidence of severe disease in RMD patients (40, 41).

Our study has some limitations. Due to its real-life nature, clinical assessment and RT-PCR timing were clinically based and differed slightly between subjects. As computed tomography was not performed, we cannot completely exclude COVID-19 pneumonia. Two fellows could not be tested for serology upon finishing their clerkship. Also, 9% of the identified RMD patients could not be reached.

In conclusion, we demonstrate that a COVID-19 outbreak can occur among HCWs and rheumatic patients, spreading over the presymptomatic stage and evolving with mild-to-moderate symptoms, delayed viral shedding, and prolonged recovery.

## Data Availability Statement

All datasets presented in this study are included in the article/Supplementary Material.

## Ethics Statement

This study was reviewed and approved by Comissão de Ética do Centro Académico de Medicina de Lisboa (reference 171/20). The patients/participants provided their written informed consent to participate in this study. Written informed consent was obtained from the individual(s) for the publication of any potentially identifiable images or data included in this article.

## Author Contributions

VR, FO-R, and JF designed the project, collected and analyzed the data, and drafted the manuscript. AC-M, PM, SB, and JS-D contacted patients and obtained the relevant clinical data. LM-G and ES-L coordinated the occupational health response. HP and JC conducted laboratory testing, including RT-PCR and serology. NK and JR overviewed clinical monitoring and data collection of healthcare workers. All members of the CHULN Rheumatology Department (collaborative group) actively participated in data acquisition. All authors have contributed to study conception and design and authors have critically reviewed the manuscript for important intellectual content and have read and approved its final version.

## Conflict of Interest

The authors declare that the research was conducted in the absence of any commercial or financial relationships that could be construed as a potential conflict of interest.
