# Supplementary material for: A COVID-19 Outbreak in a Rheumatology Department Upon the Early Days of the Pandemic
Source: Front Med (Lausanne). 2020 Sep 25;7:576162. doi: 10.3389/fmed.2020.576162 (PMC7546334; doi:10.3389/fmed.2020.576162)
Supplement: Supplementary file 1 [file Table_1.pdf]

Supplementary Table 1. Clinical, laboratory and imaging findings of healthcare workers with moderate disease.

	HCW8	HCW10	HCW11	Reference
Sex	F	F	M	-
Age	59	45	61	-
Comorbidities	UCTD, asthma, sleep apnea, dyslipidemia	Ischemic heart dx, celiac dx	Hypertension	
Smoking status	Past-smoker	Past-smoker	Past-smoker	
Concomitant therapy	MTX (15mg/w)<br>Folic acid<br>Rosuvastatin	Acetylsalicylic acid	Losartan<br>Amlodipine	
Hemoglobin (g/dL)	13.7	14.4	13.8	12.0-15.3
Leukocytes (/µL)	9,160	**3,990**	10,300	4,000-11,000
Neutrophils (/µL)	6,410	2,910	7,700	1,900-7,500
Lymphocytes (/µL)	2,040	**880**	1,680	1,000-4,800
Platelets (/µL)	436,000	224,000	**132,000**	150,000-450,000
Prothrombin time (s)	11.4	12.5	13.7	11.6
aPTT (s)	27.8	30.6	31.0	29
D-dimers (µg/mL)	**0.31**	NA	**0.27**	0-0.25
Fibrinogen (mg/dL)	**622**	NA	**474**	200-400
CRP (mg/dL)	**1.96**	0.15	**4.79**	<0.5
Procalcitonin (ng/mL)	0.04	0.03	NA	<0.5
Glucose (mg/dL)	101	89	105	67-104
Urea (mg/dL)	45	20	49	16-49
Creatinine (mg/dL)	0.63	0.57	NA	0.5-0.9
Sodium (mmol/L)	136	139	137	135-145
Potassium (mmol/L)	4.4	3.8	4.5	3.5-5.1
Chloride (mmol/L)	105	104	101	98-106
Calcium (mg/dL)	8.6	9.0	NA	8.6-10.2
Phosphate (mg/dL)	NA	3.8	NA	2.5-4.5
Magnesium (mg/dL)	2.2	2.2	NA	16.-2.6
ALT (IU/L)	**56**	9	NA	0-33
AST (IU/L)	**42**	20	NA	0-32
LDH (IU/L)	**321**	170	206	100-250
CK (IU/L)	74	23	NA	26-192
pH (arterial)	7.404	NA	7.400	7.35-7.45
pCO_2 (mmHg, arterial)	42.7	NA	41.0	35-45
pO_2 (mmHg, arterial)	88.7	NA	83.5	75-100
HCO_3 (mmol/L, arterial)	25.9	NA	24.8	22-26
Lactate (mg/dL, arterial)	10.0	NA	11.0	4.5-18
Chest X-ray	No changes	No changes	No changes	-
Treatment (duration)	HCQ 400mg/d (7d)<br>AZI 500mg/d (5d)	HCQ 400mg/d (8d)<br>AZI 500mg/d (5d)	HCQ 400mg/d (10d)<br>AZI 500mg/d (5d)	-

aPTT, activated partial thromboplastin time; ALT, alanine aminotransferase; AST, aspartate aminotransferase; AZI, azithromycin; CK, creatinine kinase; CRP, C-reactive protein; d, days; dx, disease; HCQ, hydroxychloroquine; HCW, healthcare worker; LDH, lactate dehydrogenase; NA, not available; UCTD, undifferentiated connective tissue disease.
