# Supplementary figures and images for: A COVID-19 Outbreak in a Rheumatology Department Upon the Early Days of the Pandemic
Source: Front Med (Lausanne). 2020 Sep 25;7:576162. doi: 10.3389/fmed.2020.576162 (PMC7546334; doi:10.3389/fmed.2020.576162)

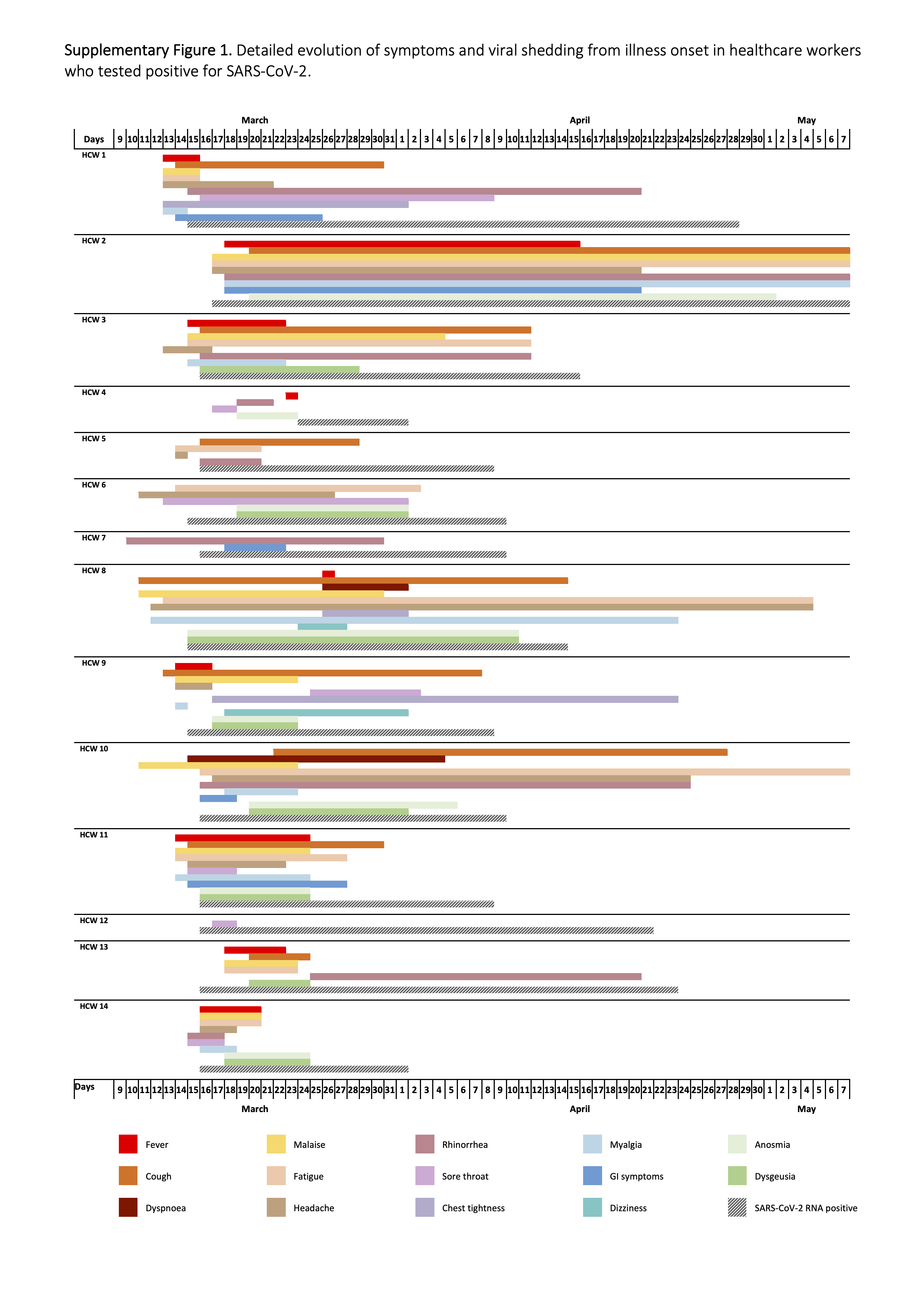

Supplement: Supplementary file 2 [file Image_1.tiff]
